# Hydrogen peroxide mediates hyperglycemia-induced invasive activity via ERK and p38 MAPK in human pancreatic cancer

**DOI:** 10.18632/oncotarget.5045

**Published:** 2015-09-05

**Authors:** Wei Li, Zhenhua Ma, Jiguang Ma, Xuqi Li, Qinhong Xu, Wanxing Duan, Xin Chen, Yunfu Lv, Shuang Zhou, Erxi Wu, Qingyong Ma, Xiongwei Huo

**Affiliations:** ^1^ Department of Hepatobiliary Surgery, The First Affiliated Hospital of Xi'an Jiaotong University, Xi'an, 710061, China; ^2^ Department of Oncology, The First Affiliated Hospital of Xi'an Jiaotong University, Xi'an, 710061, China; ^3^ Department of General Surgery, The First Affiliated Hospital of Xi'an Jiaotong University, Xi'an, 710061, China; ^4^ Department of General Surgery, People's Hospital of Hainan Province, Haikou, 570311, China; ^5^ Department of Pharmaceutical Sciences, North Dakota State University, Fargo, 58108, ND, USA

**Keywords:** diabetes, SOD2, hydrogen peroxide, MAPK pathway, pancreatic cancer invasion

## Abstract

Diabetes mellitus and pancreatic cancer are intimately related, as approximately 85% of pancreatic cancer patients suffer from glucose intolerance or even diabetes. In this study, we evaluate the underlying mechanism by which hyperglycemia modulates the invasive potential of cancer cells and contributes to their enhanced metastatic behavior. Here we show that hyperglycemia increases the hydrogen peroxide (H_2_O_2_) concentration through up-regulation of manganese superoxide dismutase (SOD2) expression, which further activates the ERK and p38 MAPK pathways, as well as the transcription factors NF-κB and AP-1, in a time-dependent manner. The invasion of pancreatic cancer cells resulting from the activation of the H_2_O_2_/MAPK axis under high glucose conditions is effectively inhibited by PD 98059 (ERK inhibitor), SB 203580 (p38 MAPK inhibitor), polyethylene glycol-conjugated catalase (PEG-CAT), or the siRNA specific to SOD2. In addition, streptozotocin-treated diabetic nude mice exhibit a stronger tumor invasive ability in renal capsule xenografts which could be suppressed by PEG-CAT treatment. Furthermore, the integrated optical density (IOD) of SOD2 and uPA stainings is higher in the tumor tissues of pancreatic cancer patients with diabetes compared with pancreatic cancer patients with euglycemia. Taken together, our results demonstrate that hyperglycemia enhances cell invasive ability through the SOD2/H_2_O_2_/MAPK axis in human pancreatic cancer. Thus, SOD2/H_2_O_2_/MAPK axis may represent a promising therapeutic target for pancreatic cancer patients combined with diabetes mellitus.

## INTRODUCTION

As the fourth leading cause of cancer death worldwide, pancreatic cancer (PC) is an aggressive malignant disease with dismal prognosis, partially due to the lack of early diagnosis and treatment options [1]. Although surgery remains the only way to cure this severe disease, more than 80% of patients cannot be considered for surgical resection at the time of initial diagnosis [2]. Even seemingly resectable PC often fails to be cured due to the systemic spread of the cancer that occurs before the operation [3]. Current treatments for inoperable patients are still limited to chemotherapy, radiation, or both [4]. Thus, more comprehensive and constructive therapeutic strategies are urgently needed.

Diabetes mellitus (DM) is one of the most common non-communicable diseases worldwide and it is likely the third modifiable risk factor for PC, after cigarette smoking and obesity [5]. Approximately 85% of patients diagnosed with PC have impaired glucose tolerance or even DM [6]. Clinical data have clearly indicated that patients with DM can aggravate the progression of PC. Patients with DM exhibit larger tumor sizes (>3 cm) and the existence of DM was reported to be independently associated with a reduced median survival [7]. Preoperative DM decreased both the rates of disease-free survival and overall survival for patients undergoing resection for PC [8]. Our own study demonstrated that DM enhances perineural invasion in PC patients and aggravates a poor prognosis [9]. However, little is known about the specific mechanism regarding the relationship between PC progression and DM.

Hyperglycemia, a typical characteristic of DM, not only induces the overproduction of reactive oxygen species (ROS), but also modulates anti-oxidative mechanisms, contributing to a pro-oxidant state [10–11]. ROS consists of a number of chemically reactive molecules derived from oxygen, including hydrogen peroxide (H_2_O_2_). As a double-edged sword, excess ROS production can kill cancer cells, whereas sublethal concentrations of ROS can stimulate tumor progression by promoting cell proliferation, survival, invasion and metastasis [12]. Our previous studies showed that high glucose (HG) can be regarded as an accelerator to increase cell proliferation through enhanced epidermal growth factor (EGF)/EGFR signaling, and as a promoter to enhance the invasive ability of PC cells [13–14]. We reasoned that the effects of DM on PC may be attributed to the induction of ROS [14]. The main antioxidant enzymes include superoxide dismutase (SOD) in the cytosol (CuZn-SOD, SOD1) and mitochondria (Mn-SOD, SOD2), which converts superoxide anion (O_2_^−^) into H_2_O_2_, as well as catalase (CAT) and glutathione peroxidase (GPX), which catabolize H_2_O_2_ into water [15]. SOD can induce the migration and invasion of cancer cells via its metabolic product H_2_O_2_ [16–17]. Meanwhile, as an H_2_O_2_ scavenger, catalase inhibits ROS-mediated tumor metastasis [12].

It has been known that ROS triggers the signaling pathways (e.g., mitogen activated protein kinase [MAPK] pathway) involved in cell migration and metastasis [18]. ROS activates members of the MAPK family including the extracellular signal-regulated kinase (ERK), c-jun NH-2 terminal kinase (JNK) and p38 MAPK, resulting in regulation of the activities of transcription factors, such as activator protein-1 (AP-1) and nuclear factor kappa B (NF-κB), which in turn modulate the expression of metastasis-related factors, including matrix metalloproteinases (MMPs) and the urokinase plasminogen activator (uPA) system. The consequence of ROS/MAPK activation finally results in local tumor invasion and distant metastasis [19].

In the current study, we investigated the production of H_2_O_2_ and the activities of the cellular antioxidant enzymes in PC cells in response to HG treatments. We also tested the hypothesis that H_2_O_2_ mediates hyperglycemia-induced activation of the MAPK signaling pathways and regulates the invasive activity of PC cells. Our findings may provide new insight into the molecular interaction between HG levels and PC and reveal a novel therapeutic strategy for PC patients who suffer from diabetes.

## RESULTS

### Effects of HG on H_2_O_2_ production and the expression of antioxidant enzymes in PC cells

**Figure 1:**
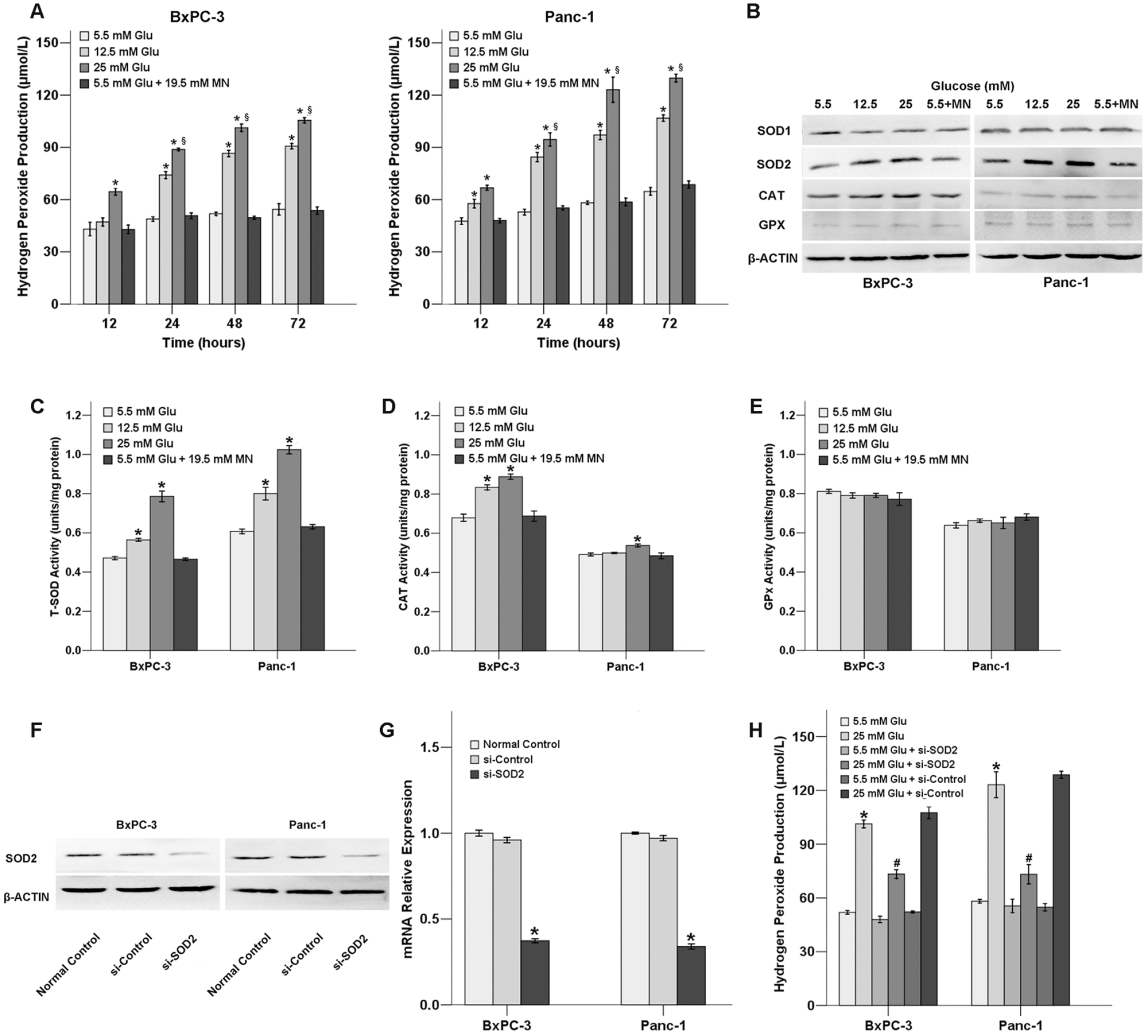
HG promotes the production of H_2_O_2_ via up-regulating SOD2 level **A.** Hydrogen peroxide production of PC cells treated with HG. BxPC-3 and Panc-1 cells were treated with the indicated concentrations of HG (5.5, 12.5, or 25 mM or 5.5 mM HG+19.5 mM mannitol), respectively, for H_2_O_2_ detection. **B.** The effect of glucose on the protein levels of antioxidant enzymes. Cancer cells were treated with different concentrations of glucose for 72 h to detect protein levels of antioxidant enzymes. **C.** The activity of the antioxidant enzymes T-SOD (total-SOD) in response to HG treatment for 72 h. **D.** The activity of the antioxidant enzymes CAT in response to HG treatment for 72 h. **E.** The activity of the antioxidant enzymes GPx in response to HG treatment for 72 h. **F.** Knockdown of SOD2 by siRNA for 48 h was confirmed using Western blot analysis. **G.** Knockdown of SOD2 by siRNA for 48 h was confirmed using QT-PCR analysis. **H.** The H_2_O_2_ level was altered in response to SOD2 knockdown after 48 h. The data are representative of 3 independent experiments. *P < 0.05 compared to the 5.5 mM glucose group; ^§^*P* < 0.05 compared to the 25 mM glucose group at 12 h; ^#^*P* < 0.05 compared to the 25 mM glucose + si-control group.

To explore the possible relationship between HG and oxidative stress, we first examined the effects of HG on H_2_O_2_ production in PC cells. Our results show that the levels of H_2_O_2_ in BxPC-3 and Panc-1 cells are up-regulated in response to high glucose concentrations in comparison to normal physiological glucose level (5.5 mM glucose) in both time-dependent and dose-dependent manners. Additionally, the HG-induced change in H_2_O_2_ production was not attributable to the osmolarity effects on the cancer cells because the addition of mannitol, an osmosis regulator, did not affect the level of H_2_O_2_ (Figure 1A).

Next, the protein expressions and activities of antioxidant enzymes in the PC cells exposed to HG were evaluated using Western blot analysis and antioxidant enzyme activity assay. As shown in Figure 1B, the protein levels of SOD2 and CAT were up-regulated in response to increasing concentrations of HG. However, the expression of SOD1 and GPX protein levels was not influenced by HG. Treatment with mannitol did not affect the expression of the antioxidant enzymes. HG condition could also influence the activity of the antioxidant enzymes SOD and CAT, and this trend was consistent with the results from the protein expression analysis (Figure 1C–1E). All of the tested antioxidant enzymes exhibited cytoplasmic localization in both the BxPC-3 and Panc-1 cancer cells (Supplementary Figures S1).

### SOD2 is involved in the HG-induced up-regulation of H_2_O_2_ production

To further examine whether SOD2 could influence H_2_O_2_ production under HG conditions, we used SOD2 siRNA to knock down SOD2 expression in both BxPC-3 and Panc-1cancer cells and then examined the H_2_O_2_ levels (Figure 1F, 1G). Our results show that the increased H_2_O_2_ production of the PC cells in the presence of HG was diminished when SOD2 was knocked down, indicating that the HG-induced H_2_O_2_ level is dependent on SOD2 (Figure 1H).

### HG activates the ERK and p38 MAPK signaling pathways via the production of H_2_O_2_

**Figure 2:**
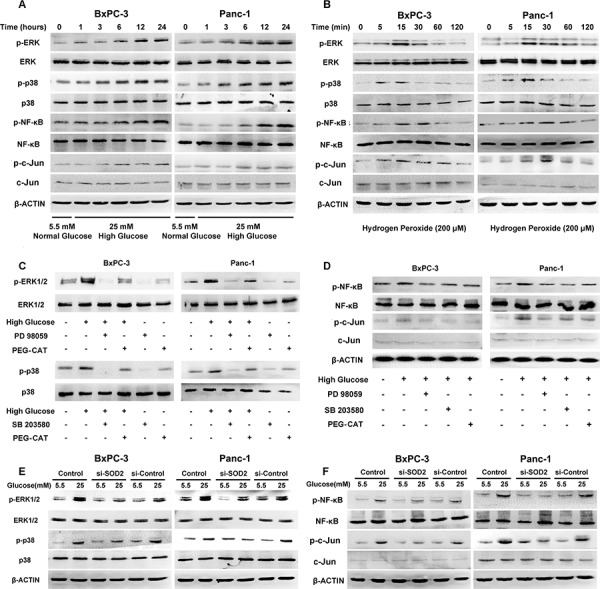
HG activates MAPK pathways and the NF-κB and AP-1 transcription factors via the production of H_2_O_2_ in BxPC-3 and Panc-1 cells **A.** The effect of 25 mM glucose on the phosphorylation of ERK, p38 MAPK, NF-κB and c-Jun. PC cells were treated with 25 mM glucose for the indicated times. Phosphorylation of ERK, p38 MAPK, NF-κB and c-Jun were determined using Western blot analysis. **B.** The effect of H_2_O_2_ (200 μM) on the phosphorylation of ERK, p38 MAPK, NF-κB and c-Jun. PC cells were treated with 200 μM H_2_O_2_ for the indicated times. Phosphorylation of ERK, p38 MAPK, NF-κB and c-Jun were determined using Western blot analysis. **C.** The effect of MAPK pathway inhibitors on the phosphorylation of ERK and p38. **D.** The effect of MAPK pathway inhibitors on the phosphorylation of NF-κB and c-Jun. BxPC-3 and Panc-1 cells were treated with the selective MAPK pathway inhibitors PD 98059 (50 μM) and SB 203580 (20 μM), as well as PEG-CAT (1000 U/ml) in the presence or absence of high glucose concentrations. The phosphorylation of ERK, p38 (C), NF-κB and c-Jun (D) were analyzed using Western blot analysis for 24 h. **E.** The effect of SOD2 knockdown on the phosphorylation of ERK and p38 in the presence or absence of high glucose concentrations. **F.** The effect of SOD2 knockdown on the phosphorylation of NF-κB and c-Jun in the presence or absence of high glucose concentrations. After the BxPC-3 and Panc-1 cells were transfected with siRNAs for 48 h, the phosphorylation levels of ERK, p38 (E), NF-κB and c-Jun (F) were determined using Western blot analysis.

To determine whether HG could influence the activation of MAPK signaling pathways, we analyzed the expression of ERK, p38 MAPK as well as the related transcription factors using Western blot analysis. As shown in Figure 2A, HG treatment induced the phosphorylation of ERK and p38 MAPK, as well as the phosphorylation of the transcription factors NF-κB and c-Jun, in a time-dependent manner in the PC cells. The increased phosphorylation levels of ERK and p38 MAPK were detected after 1 h of stimulation with HG and remained at high levels until 24 h, while the activation of p-NF-κB and p-c-Jun began after 3 h of HG stimulation.

To investigate whether the activation of the MAPK signaling pathway was related with the production of H_2_O_2_, we directly treated BxPC-3 and Panc-1 cancer cells with H_2_O_2_. To determine the best intervention concentration of H_2_O_2_ to use on the PC cells, we cultured BxPC-3 and Panc-1 cells in media containing increasing concentrations (0 – 800 μM) of H_2_O_2_ and the effects on cell proliferation were determined using an MTT assay. The results show that H_2_O_2_ induces cell proliferation in BxPC-3 cells in a dose-dependent manner after incubation for 24, 48, or 72 h with 0 to 200 μM H_2_O_2_; however, H_2_O_2_ was cytotoxic at concentrations above 200 μM. A similar effect of H_2_O_2_ was also observed in the Panc-1 cancer cells (Supplementary Figures S2A). Therefore, a treatment concentration of 200 μM H_2_O_2_ was used in the subsequent experiments. As shown in Figure 2B, H_2_O_2_ activated ERK and p38 MAPK in a time-dependent manner, with peak activation between 5 and 15 min. Meanwhile, H_2_O_2_ treatment also led to an increase in the phosphorylation level of NF-κB and c-Jun, with a peak between 15 and 30 min.

We next treated both BxPC-3 and Panc-1 cells with the ERK inhibitor, PD 98059, or the p38 MAPK inhibitor, SB 203580, and tested for the best intervention concentrations. The results demonstrate that both inhibitors suppress cells’ proliferation in a concentration-dependent manner in both cell lines. For cells under treatment for 72 h, the IC_50_ of PD 98059 in both PC cell lines was approximately 50 μM, whereas the IC_50_ of SB 203580 in BxPC-3 and Panc-1 cancer cells was approximately 20 μM (Supplementary Figure S2B, S2C). To further assess whether the HG-induced H_2_O_2_ production was involved in the MAPK activation, PC cells were treated with both HG and polyethylene glycol-conjugated catalase (PEG-CAT), which is able to prolong the circulatory half-life of the native CAT enzyme and enhances its intracellular access. Interestingly, we found that PEG-CAT diminished the HG-induced ERK and p38 MAPK phosphorylation (Figure 2C). Treating the PC cells with PEG-CAT and PD 98059 or SB 203580 also diminished the phosphorylation levels of NF-κB and c-Jun, indicating that H_2_O_2_ and the MAPK signaling pathway mediates the activation of these transcription factors (Figure 2D). Furthermore, as shown in Figure 2E, the phosphorylation levels of ERK and p38 MAPK were significantly decreased following SOD2 knockdown, indicating that the SOD2-induced H_2_O_2_ level is intimately related to the MAPK pathway. The phosphorylation levels of NF-κB and c-Jun were also diminished in the si-SOD2 group (Figure 2F).

### H_2_O_2_ production is required for the HG-induced up-regulation of uPA

**Figure 3:**
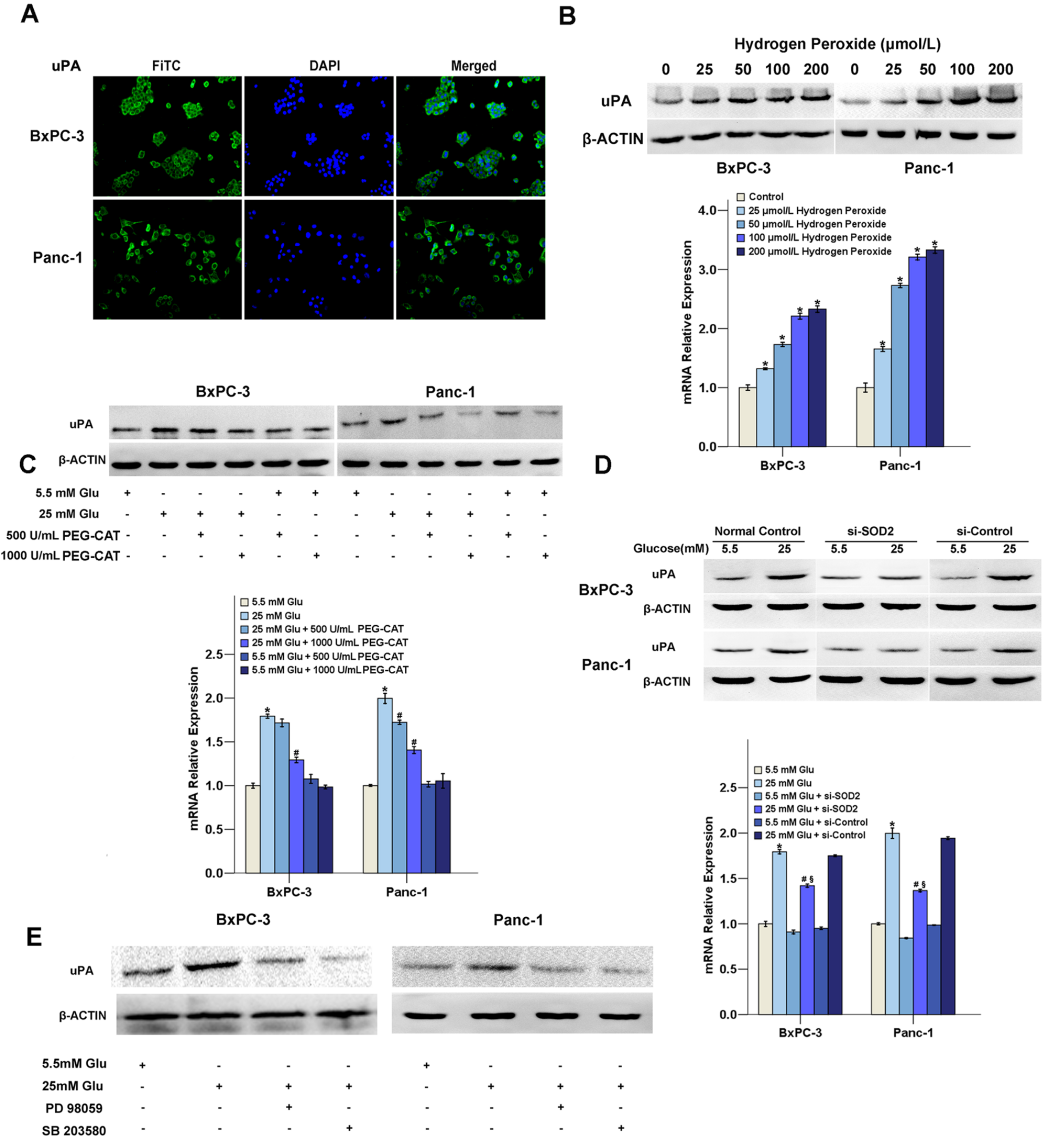
Effect of H_2_O_2_ on uPA expression **A.** The subcellular localization of uPA. PC cells were labeled with a fluorescence-conjugated uPA specific antibody (green) (×200). The nuclei were stained with DAPI. uPA was localized exclusively in the cytoplasm. **B.** BxPC-3 and Panc-1 cells were treated with different concentrations of H_2_O_2_ (0, 25, 50, 100 or 200 μM) for 48 h for mRNA and protein detection. **C.** The regulation on uPA expression in response to PEG-CAT treatment (500 or 1000 U/ml). **D.** The regulation on uPA expression in response to SOD2 knockdown. **E.** The regulatioin on uPA expression in response to and MAPK pathway inhibitors. The data are representative of 3 independent experiments. *P < 0.05 compared to the 5.5 mM glucose group; ^#^*P* < 0.05 compared to the 25 mM glucose group; ^§^*P* < 0.05 compared to the 25 mM glucose + si-control group.

Our previous study demonstrated that HG could promote the invasive ability of PC cells through the up-regulation of the metastasis-related factor uPA [14]. To examine whether the HG-induced uPA expression is related to the H_2_O_2_ level, H_2_O_2_, PEG-CAT and si-SOD2 were used to test the expression of uPA. As illustrated in Figure 3, the expression of uPA that is located in the cytoplasm (Figure 3A), was increased following the addition of H_2_O_2_ in a dose-dependent manner (Figure 3B). PEG-CAT cleared the produced H_2_O_2_, and si-SOD2 decreased the production of H_2_O_2_, both of PEG-CAT and si-SOD2 down-regulated the expression of uPA under HG conditions in PC cells (Figure 3C, 3D). In addition, both PD 98059 and SB 203580 could decrease the expression of uPA in PC cells under HG conditions, indicating that the activation of the ERK and p38 MAPK signaling pathway is involved in HG-induced uPA expression (Figure 3E).

### Increased H_2_O_2_ production and activation of the ERK and p38 MAPK signaling pathways are involved in HG-induced cell invasion

**Figure 4:**
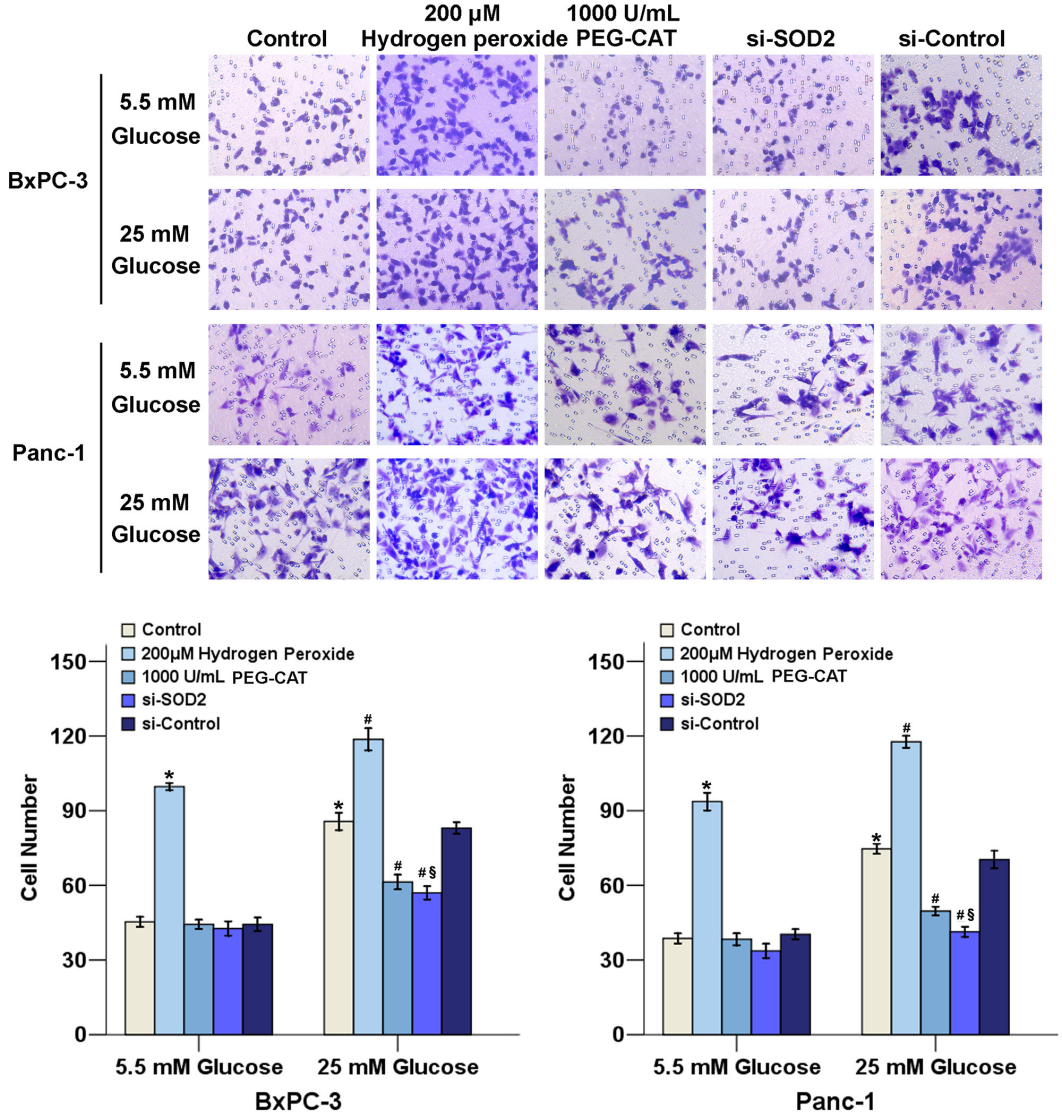
The effect of H_2_O_2_ on the cancer cell's invasive ability under HG conditions PC cells in DMEM medium containing 1% FBS were seeded in the matrigel-coated Transwell upper chambers. 20% FBS was used as chemoattractant. Cancer cells were treated with 200 μM H_2_O_2_, 1000 U/L PEG-CAT or si-SOD2 in the presence or absence of high glucose concentrations. After 48 h, the cells on the upper surface of the filters were removed; the filters were then stained with crystal violet. The number of migrated cells was quantified by counting the cells from 10 random fields at ×200 magnification. The data are representative of 3 independent experiments. *P < 0.05 compared to the 5.5 mM glucose group; ^#^*P* < 0.05 compared to the 25 mM glucose group, ^§^*P* < 0.05 compared to the 25 mM glucose + si-control group.

**Figure 5:**
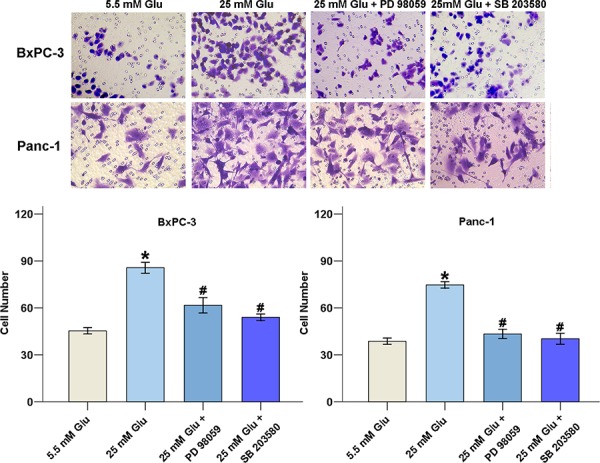
MAPK activation modulates PC progression BxPC-3 and Panc-1 cells were exposed to the MAPK pathway inhibitors PD 98059 (50 μM) and SB 203580 (20 μM) for 48 h, the number of migrated cells was quantified by counting the number of cells from 10 random fields at ×200 magnification. The data are representative of 3 independent experiments. *P < 0.05 compared to the 5.5 mM glucose group; ^#^*P* < 0.05 compared to the 25 mM glucose group.

To explore whether the H_2_O_2_ level is involved in the HG-induced cell invasive ability, PC cells were treated with H_2_O_2_, PEG-CAT, and si-SOD2, and the invasive abilities of the cells were examined under different glucose conditions. The results show that both HG and H_2_O_2_ increased the invasion rates of cancer cells. HG-induced cell invasion decreased when H_2_O_2_ was eliminated by either PEG-CAT or si-SOD2 treatment (Figure 4). Furthermore, both PD 98059 and SB 203580 could decrease PC cells invasion under HG conditions, indicating that the activation of the ERK and p38 MAPK signaling pathways is involved in HG-induced PC cell invasion (Figure 5).

### PEG-CAT inhibits tumor invasion in streptozotocin (STZ)-treated nude mice

**Figure 6:**
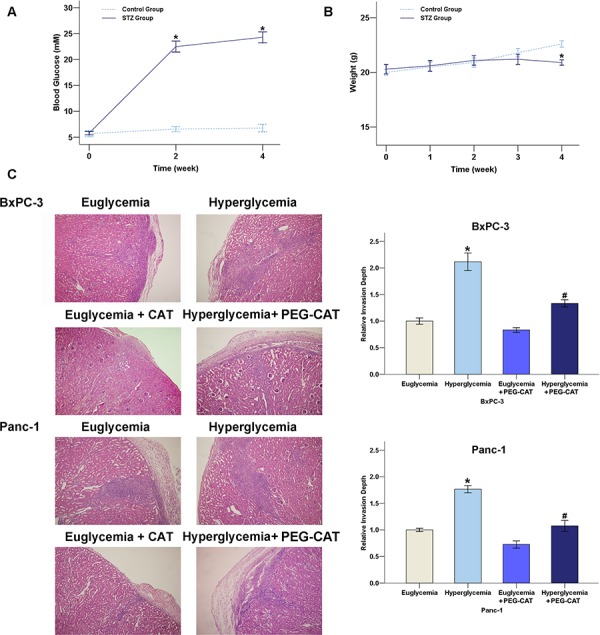
Effect of the hyperglycemia/H_2_O_2_ axis on the invasion of PC in nude mice **A.** Blood glucose in STZ-treated mice (*n* = 12). **B.** Body weight in STZ-treated mice (*n* = 12). **C.** The H&E staining results showed that the invasion ability of the PC cells was enhanced in the renal capsule xenografts of the hyperglycemic mice and this increase could be strongly suppressed by repeated injections of PEG-CAT. *P < 0.05 compared to euglycemia group; ^#^*P* < 0.05 compared to hyperglycemia group.

To determine the efficacy of different drugs against transplantation-established human tumor xenografts in the athymic nude mice, we used a subrenal capsular assay. STZ is a chemical which is commonly used to induce experimental diabetes in animals [20]. The characteristics of the STZ-treated nude mice used in this study were summarized in Figure 6. The fasting blood glucose levels were significantly increased from 2 weeks to 4 weeks after STZ injection (Figure 6A) and the body weight of the nude mice was reduced at 4 weeks after STZ injection (Figure 6B).

In the subrenal capsular assay, cancer cells were maintained in a discrete, solid structure under the kidney capsule and were able to invade into the kidney parenchyma, identified by detection of the tumor cells surrounding renal glomeruli and tubules. As shown by the H&E staining results in Figure 6C, the invasive ability of both the BxPC-3 and Panc-1 cells were strongly enhanced in the DM renal capsule xenograft model and this increase could be suppressed by PEG-CAT treatment. Taken together, these results indicate that the hyperglycemia-induced increase of the level of H_2_O_2_ is involved in the acceleration of tumor invasion.

### The expression of SOD1, SOD2, and uPA in pancreatic ductal carcinoma specimens

Of all 417 cases, 149 (35.73%) had hyperglycemia (new-onset diabetes and uncontrolled diabetes). The remaining 268 cases (euglycemia group) included controlled diabetic or non-diabetic cases. The hyperglycemia and euglycemia groups were similar in terms of age, sex, and body mass index (BMI). Among the 149 hyperglycemia cases, 47 had a history of diabetes, compared with 14 of 268 euglycemia cases (*P* < 0.001, Chi-square test).

**Figure 7:**
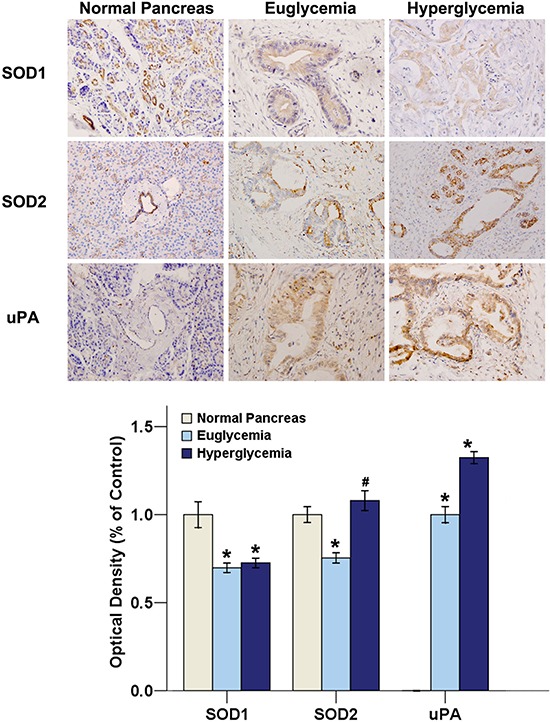
Hyperglycemia promotes SOD2 and uPA expressions in human PC tissues Sixty-five patients (19 specimens in the hyperglycemia group and 46 specimens in the euglycemia group) who received a radical curative pancreatic operation with a pathologic diagnosis and 8 normal pancreases were investigated. The SOD1, SOD2 and uPA immuno-stainings were analyzed by Image-Pro Plus software. Original magnification ×400. *P < 0.05 as compared with normal pancreas group. ^#^*P* < 0.05 as compared with euglycemia group.

We further investigated the change in the expression levels of the SOD and uPA in human PC specimen in the euglycemia and hyperglycemia groups. As shown in Figure 7, the majority of SOD1, SOD2, and uPA staining was located at the cytoplasm. There is a decreased staining intensity of SOD1 and SOD2 in PC specimens when compared with normal pancreas. Little or no uPA immunoreactivity was observed in normal pancreatic tissues. The SOD2 and uPA stainings in the cytoplasm of the cancer cells was significantly stronger in the hyperglycemia group than in the euglycemia group, indicating that hyperglycemia is able to increase the expression of SOD2 and uPA protein levels. The percentage of positive SOD2 and uPA staining cancer cells were significantly higher in the hyperglycemia group than in the euglycemia group. However, there was little difference in SOD1 expression between hyperglycemia group and euglycemia group.

## DISCUSSION

Due to the high rate of locally deteriorated or metastatic disease at the time of diagnosis and the lack of effective medical therapy, PC has been considered one of the most lethal malignant diseases. In recent years, although the largest improvements in survival have been made for a number of other cancers, PC still shows the least improvement [1]. It has been estimated that the mortality rates of cancers of the lung, colon, breast and prostate will decline due to the combined effects of widespread screening, smoking cessation and more effective therapy, and PC may become the leading cause of cancer-related deaths in the USA by 2050 [21]. Therefore, the exploration of risk factors, metastatic mechanisms and therapeutic targets for PC has become hot topics in recent years.

DM, which is a very common metabolic disorder characterized by hyperglycemia that eventually affects all of the systems in the body, has been postulated to be both an independent risk factor and a consequence for PC in recent years [22]. On one hand, a large number of epidemiological studies have demonstrated that DM increases an individual's risk of developing PC [23] and anti-diabetic drugs can be used to prevent and treat PC [24]. On the other hand, DM may be a consequence of PC because there is an increased prevalence of diabetes in PC patients and DM may be resolved after surgery (pancreaticoduodenectomy) [25]. However, little is known about the specific mechanism underlying this linkage.

Our data show that the hyperglycemia-induced invasive ability of PC cells could be attributed to the production of ROS. There are 6 major pathways that contribute to the production of ROS under HG conditions: glyceraldehyde autoxidation, PKC activation, glycation, sorbitol metabolism, the hexosamine pathway, and oxidative phosphorylation [26]. ROS can be considered as a double-edged sword in cancer cells; excess ROS production can kill cells, whereas sublethal concentrations of ROS can promote cell progression [12]. Our results indicate that high glucose resulted in an increase in the level of H_2_O_2_ to a non-toxic level in PC cells; treatment with H_2_O_2_ promoted cancer cell proliferation when its concentration was below 200 μM, but H_2_O_2_ concentrations above 200 μM were toxic to the cancer cells. Recently, Ikemura et al. [27] showed that there were larger and more tumor metastatic colonies in the lung and liver of the STZ-treated mice and injections of PEG-CAT could effectively inhibit tumor metastasis. They concluded that the increased oxidative stress, not a high blood glucose level, could accelerate tumor metastasis in hyperglycemic mice. Additionally, the hyperglycemia-induced increase in ROS also resulted in the up-regulation of adhesion molecules expression, through which tumor cells adhere to endothelial cells to form metastatic colonies [28–29]. H_2_O_2_ is diffusible and thus capable of traveling across plasma membranes into the extracellular space to exert both paracrine and autocrine roles. In our *in vitro* study, we observed that H_2_O_2_ could increase the invasive ability of the PC cells, whereas treatment with either PEG-CAT, which cleared the produced H_2_O_2_, or si-SOD2, which decreased the production of H_2_O_2_, could terminate this effect. Additionally, in our *in vivo* study, we demonstrated that a single injection of STZ could lead to a reduction in body weight and an increase in the fasting blood glucose level of nude mouse. Hyperglycemic conditions enhanced the invasive ability of both BxPC-3 and Panc-1 cells in renal capsule xenografts and this effect might be attributed to the production of H_2_O_2_, as injection of PEG-CAT effectively inhibited tumor invasion in the STZ-treated mice. In addition, hyperglycemia is correlated with increased expression levels of SOD2 and uPA in the tissue specimens obtained from PC patients. As our previous study demonstrated that DM enhances perineural invasion in PC patients [9], we assumed that this enhancement might be related with HG-induced H_2_O_2_ production.

Cellular SOD converts intracellular superoxide radicals into H_2_O_2_ that is cleared by GPX and CAT and converted into water. It has been shown that the SOD activity was much higher in both diabetic patients [30–31] and STZ-treated/alloxan-induced diabetic mice compared to those with normal blood glucose [20, 32]. SODs are a family of antioxidant enzymes responsible for the detoxification of superoxide anion. SOD1 is located in the cytoplasm, while SOD2 is in the mitochondria [15]. In the current study, we found that HG increases SOD2, but not SOD1 expression, which might indicate that hyperglycemia preferentially affect H_2_O_2_ production in mitochondria. In addition, we also showed that SOD-induced H_2_O_2_ production promoted tumor cell invasion; while si-SOD2 and PEG-CAT treatment inhibits these effects. In recent years, increased SOD2 levels have been associated with poor prognosis and resistance to therapy of several tumors in the central nervous system, gastrointestinal tract, head and neck [33]. Toh et al. [34] reported 2.19- and 3.72-fold increases in SOD2 mRNA expression relative to normal tissue in gastric and colorectal cancers, respectively. Lewis et al. [35] indicated that nude mice that were injected with human PC cells occasionally developed intra-abdominal metastatic deposits and ascites that were associated with an increase in SOD2 activity. The A allele of the *SOD2* − 1221G > A genotype was associated with a higher risk of pancreatic cancer among individuals with a low dietary vitamin E intake [36]. However, there are also many reports that SOD2 has characteristics of tumor suppressor and can inhibit cancer development and progression [37]. Hempel et al. [38] concluded that there is a dichotomous role for SOD2 during tumorigenisis. SOD2 may act as a tumor suppressor during the initial onset/proliferative stage of tumor initiation, yet once the tumor progresses to a more aggressive and invasive phenotype, SOD2 levels appear to positively correlate and contribute to enhanced metastatic behavior of cancer cells. Our results support the concept that metastatic disease is associated with changes in the content and activity of the antioxidant enzymes [35]. Additionally, In agreement with other reports [31–39], the activity of CAT was also found to be slightly increased under HG conditions. This may be attributed to the fact that persistent hyperglycemia leads to increased H_2_O_2_ levels, which eventually results in the induction of CAT activity.

The MAPK signaling pathways are important signaling cascades downstream of ROS that are involved in tumor migration and invasion [18]. Cheng et al. [40] observed that H_2_O_2_ is a mediator of the epidermal growth factor (EGF)-mediated activation of the p38 MAPK pathway and cell invasion in ovarian cancer cells. Lee et al. [41] also showed that hepatocyte growth factor (HGF) regulates H_2_O_2_ production which further activates the ERK pathway and regulates uPA production, eventually increasing the invasive potential of stomach cancer cells. Our results show that HG could promote the activation of p-ERK, p-p38, p-NF-κB and p-c-Jun via H_2_O_2_. After suppressing the MAPK signaling pathway by treatment with PD 98059 or SB 203580, the invasive ability of PC cells decreased. Additionally, the down-regulation of H_2_O_2_ by either PEG-CAT or si-SOD2 inhibits the activation of the MAPK signaling pathways and tumor invasion, which indicates that H_2_O_2_ is the key factor that mediated HG-induced PC metastasis.

## MATERIALS AND METHODS

The study protocol and consent forms conform to the Declaration of Helsinki and were approved by the Ethical Review Board (ERB) Committee (The First Affiliated Hospital of Medical College, Xi'an Jiaotong University, China) and the written informed consent was obtained from all participants.

### Collection of tissues and immunohistochemistry

From January 2007 to July 2014, 520 pancreatic tumors were subjected to clinical examination at the First Affiliated Hospital, Xi'an Jiaotong University, China. Among them, 417 cases were diagnosed with pancreatic ductal carcinoma excluding islet cell carcinoma, intraductal papillary mucinous tumor and acinar cell carcinoma. The 417 pancreatic tumors were divided into two groups according to their fasting blood glucose levels (an average long-term glucose level for patients with a history of diabetes mellitus and an average three-day glucose level after hospitalization): (1) euglycemia or normal (no diabetic or controlled diabetic cases) and (2) hyperglycemia or high (new-onset diabetes and uncontrolled diabetes). Most PC patients were diagnosed at the advanced-stage and not suitable for surgical resection. Therefore, only sixty-five patients who received a radical curative pancreatic operation with a pathologic diagnosis and 8 normal pancreases (also from the First Affiliated Hospital, Xi'an Jiaotong University) were included in this study. Formalin fixed and paraffin embedded PC tissue samples from the above fifty-nine patients were used for the immunohistochemistry test.

The tissue sections were incubated with primary antibodies (anti-SOD1, anti-SOD2 and anti-uPA, 1:50) overnight at 4°C and incubated with the appropriate biotinylated secondary antibody for 30 min at room temperature. After rinsing, the results were visualized using diaminobenzidine (DAB) and the slides were counterstained with hematoxylin. The densitometry analysis of immunohistochemical staining was performed using the Image-Pro Plus 6.0 software.

### Cell culture and reagents

The human PC cell lines, BxPC-3 and Panc-1, were obtained from the American Type Culture Collection (Manassas, VA, USA). The cells were cultured in Dulbecco's Modified Eagle's Medium (DMEM) supplemented with 10% dialyzed heat-inactivated fetal bovine serum, 100 U/ml penicillin and 100 μg/ml streptomycin in a 95% air/5% CO_2_ humidified atmosphere at 37°C. Catalase derivative conjugated to polyethylene glycol (PEG-catalase) and streptozotocin (STZ) were acquired from Sigma Aldrich (St. Louis, MO, USA). The ERK inhibitor PD 98059 and the p38 MAPK inhibitor SB 203580 were obtained from Sigma Chemical Co. Primary antibodies against SOD1, SOD2, CAT, GPX1 and uPA were procured from Santa Cruz Biotechnology (Santa Cruz, CA, USA). The anti-ERK, anti-phospho-ERK (Thr202/Tyr204), anti-p38 MAPK, anti-phospho-p38 MAPK (Thr180/Tyr182), anti-NF-κB, anti-phospho-NF-κB p65 (Ser468), anti-c-Jun and anti-phospho-c-Jun (Ser63) antibodies were obtained from Cell Signaling Technology (Beverly, MA, USA).

### Hydrogen peroxide assay

The level of intracellular H_2_O_2_ was measured using hydrogen peroxide assay kit according to the manufacturer's instructions. In this kit, the ferrous ions Fe^2+^ were oxidized to ferric ions Fe^3+^ by H_2_O_2_. The ferric ions further formed a complex with the indicator dye xylenol orange and produced a visible purple-colored complex that could then be measured using a microplate reader at a wavelength of 560–590 nm (Bio-Rad, CA, USA).

### Antioxidant enzyme activities

The bioactivity of SOD was measured using a total SOD activity assay kit (Beyotime, Jinan, China). This assay is based on the ability of SOD to inhibit the formation of water-soluble formazan dye that results from the reaction between superoxide anion and WST-1. The absorbance at a wavelength of 450 nm was measured using a microplate reader. The activity of total SOD was calculated according to the manufacturer's instructions.

The CAT activity was detected using a catalase analysis kit (Beyotime, Jinan, China). Briefly, the cell lysates were treated with excess H_2_O_2_ that was decomposed by CAT for the indicated times. Afterwards, the remaining H_2_O_2_ was coupled to a substrate and was catalyzed by peroxidase to generate the red product that could be detected using a microplate reader at a wavelength of 520 nm. Catalase activity was then calculated from the assay results.

The activity of GPX was examined using a total glutathione peroxidase assay kit (Beyotime, Jinan, China). Briefly, GPX was indirectly measured by evaluating the level of NADPH oxidation in a coupled assay system, containing glutathione, glutathione reductase and cumene hydroperoxide as the substrate, using a microplate reader at a wavelength of 340 nm. GPX (1 unit) was defined as the amount of enzyme that oxidized 1 nmol of NADPH in one minute.

### MTT assay

BxPC-3 and Panc-1 cells were seeded in 96-well plates at a density of 5,000 to 10,000 cells per well and were incubated overnight in medium containing 10% FBS. The cells were then treated with increasing concentrations of PD 98059, SB 203580, or H_2_O_2_. After incubation for 24, 48, or 72 h at 37°C, the MTT reagent was added to each well and was incubated at 37°C for 4 h. DMSO was then added to each well and the optical density (OD) value at 490 nm was determined using a spectrophotometer (Bio-Rad, CA, USA). The results are presented as the percentages relative to the controls. The proliferation rate was defined as = OD_sample_/OD_control_ × 100%.

### Transwell matrigel invasion assay

The invasive ability of the PC cells was analyzed using matrigel invasion chambers. The 8.0 μm pore inserts were coated with 30 μl of matrigel. Cell suspensions (5 × 10^4^) were added to the upper chambers in DMEM containing 1% FBS. 500 μl of DMEM containing 20% FBS was placed in the lower chambers. The matrigel invasion chamber was then incubated for 48 h in a humidified tissue culture incubator. The non-invading cells were removed from the upper surface by scraping with a wet cotton swab. After rinsing with PBS, the filter was fixed and stained with crystal violet. The invasion ability was determined by counting the stained cells on the bottom surface.

### Real-time quantitative PCR (QT-PCR)

Total RNA was extracted from the PC cells using the Fastgen200 RNA isolation system (Fastgen, Shanghai, China). Total RNA was reverse-transcribed into cDNA using the PrimeScript RT reagent Kit (TaKaRa, Dalian, China). The PCR reactions consisted of 30 s at 95°C, followed by 40 cycles of 95°C for 5 s, 60°C for 30 s and 72°C for 30 s. The relative gene expression was calculated using the previously described 2^−ΔΔCt^ method [42]. The primer sequences were as follows:

SOD2-F: 5′-CAC CAC AGC AAG CAC CAC-3′.

SOD2-R: 5′-GTT CTC CAC CAC CGT TAG G-3′.

uPA-F: 5′-TAA GAG CTG GTG TCT GAT TG-3′.

uPA-R: 5′-TTG GAT GAA CTA GGC TAA AA-3′.

β-actin-F: 5′-GAC TTA GTT GCG TTA CAC CCT TTC T-3′.

β-actin-R: 5′-GAA CGG TGA AGG TGA CAG CAG T-3′.

### Western blot analysis

Proteins were electrophoretically resolved on a denaturing SDS-polyacrylamide gel and were electrotransferred onto polyvinylidene difluoride membranes. The membranes were initially blocked with 5% nonfat dry milk in Tris-buffered saline (TBS) for 2 h and then probed with each primary antibody. After co-incubation with the primary antibodies at 4°C overnight, the membranes were blotted with the secondary antibody for 2 h at 37°C. The results were visualized using the ECL Western blotting substrate and were photographed by GeneBox (SynGene).

### RNAi transfections

siRNA against SOD2 (SOD2-Homo-429: 5′-GGU GGU CAU AUC AAU CAU ATT-3′, 5′-UAU GAU UGA UAU GAC CAC CTT-3′) and a negative control siRNA (NC: 5′-UUC UCC GAA CGU GUC ACG UTT-3′, 5′-ACG UGA CAC GUU CGG AGA ATT-3′) were purchased from GenePharm (Shanghai, China).

### Subrenal capsular assay and histopathology

Five week old BALB/c athymic nude mice were purchased from Shanghai Experimental Animal Center. Animal care and experiments were carried out in accordance with the guidelines of Institutional Animal Care and Use Committee at Xi'an Jiaotong University. For DM group, BALB/c athymic nude mice received an intraperitoneal injection of STZ at a dose of 175 mg/kg body weight.

The subrenal capsular assay, a rapid *in vivo* test system for observing tumor proliferation and invasion, was established to test the efficacy of different drugs against transplantation-established human tumor xenografts in the athymic nude mice (*n* ≥ 6 for each group).

Two weeks after STZ injection, each mouse was used for the subrenal capsule assay. The BxPC-3 and Panc-1 cell lines were used to create xenografts in the athymic nude mice. After anesthetization, the left kidney of each mouse was exposed and the same number of cells (1 × 10^8^) was injected under the left renal capsule in a total volume of 25 (l. Three days later, PEG-CAT was intraperitoneally injected into a group of mice at a dose of 1000 units/d. The cells maintained a discrete, solid structure under the kidney capsule, and 3-dimensional growth was observed microscopically and directly quantified by changes in the average diameter. After inoculation with the tumor cells, the mice were sacrificed on day 14 and their left kidneys were excised.

The left renal capsule, as well as the xenografts, were fixed in 10% neutral buffered formalin and were processed in paraffin. Sections were cut using a microtome and were mounted on glass slides. After dewaxing, the sections were hydrated in graded alcoholic solutions and distilled water. Routine H&E staining was performed.

### Statistical analysis

Statistical analyses were performed using SPSS software (version 17.0, SPSS Inc., Chicago, USA). Data were presented as the means ± SEM of three replicate assays. Differences between the groups were analyzed by analysis of variance (ANOVA). Statistical significance was set at *P* < 0.05. All experiments were repeated independently at least three times.

## CONCLUSION

The current study demonstrates that H_2_O_2_ contributes to the SOD2-induced invasion in PC cells *in vitro* by modulating the expression of the metastasis-related factor uPA through the activation of the ERK and p38 MAPK signaling pathways. This study also provides evidence that accelerated tumor invasion in hyperglycemic nude mice is due to the increased production of H_2_O_2_, which can be effectively inhibited by repeated injections of PEG-CAT. In addition, hyperglycemia is correlated with increased expression levels of SOD2 and uPA in the tissue specimens obtained from PC patients. These findings suggest that blocking the SOD2/H_2_O_2_/MAPK signaling system may provide a novel strategy for the treatment of PC accompanied by DM.

## SUPPLEMENTARY MATERIALS FIGURES


