# Supplementary material for: Hydrogen peroxide mediates hyperglycemia-induced invasive activity via ERK and p38 MAPK in human pancreatic cancer
Source: Oncotarget. 2015 Sep 5;6(31):31119–33. doi: 10.18632/oncotarget.5045 (PMC4741592; doi:10.18632/oncotarget.5045)
Supplement: Supplementary file 1 [file oncotarget-06-31119-s001.pdf]

SUPPLEMENTARY FIGURES

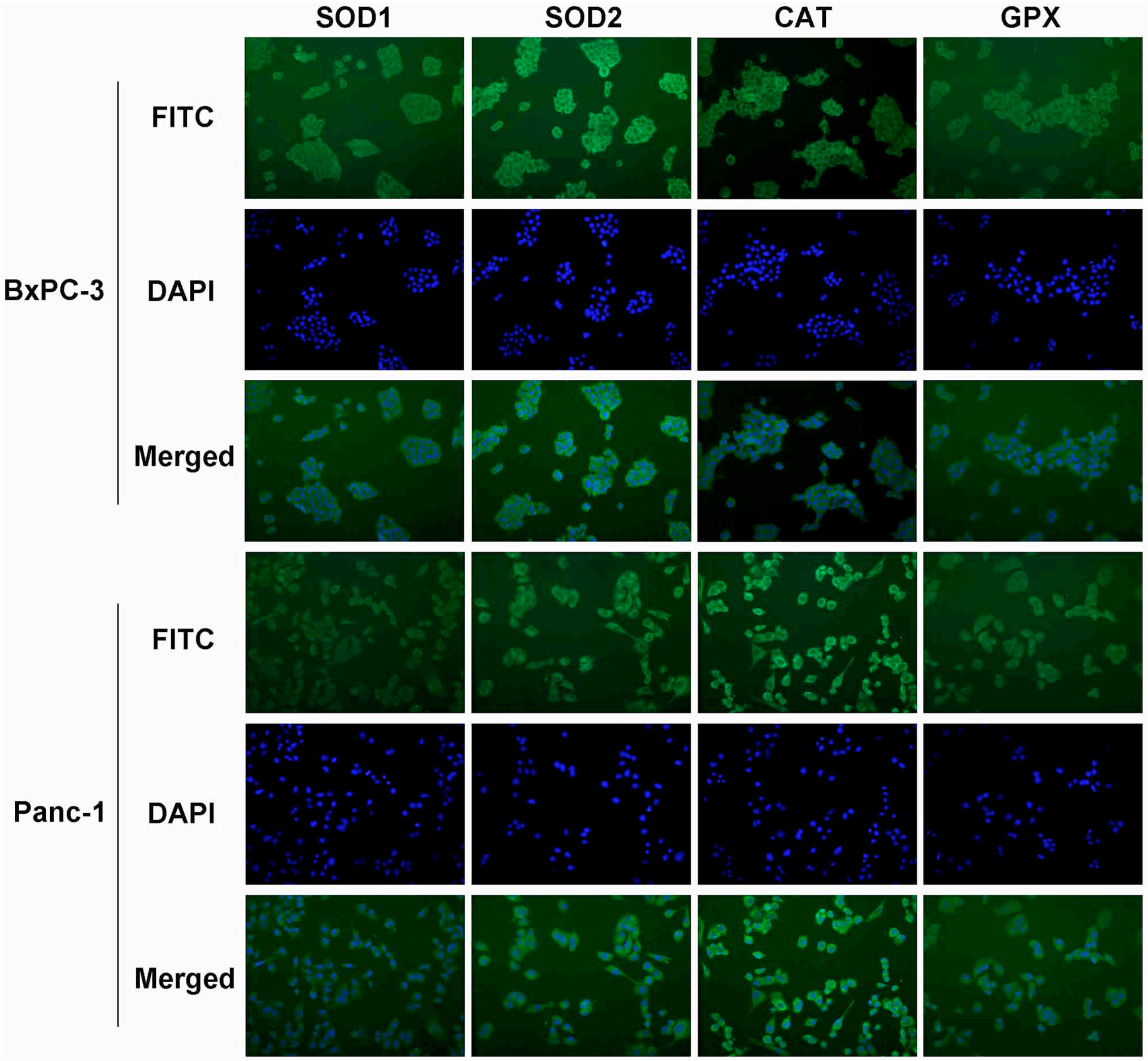

Supplementary Figure S1: Expression of antioxidant enzymes in BxPC-3 and Panc-1 cells. Cells were labeled with fluorescence-conjugated SOD1/SOD2/CAT/GPX specific antibody (green) (×200). Nucleus was stained with 4′,6-diamidino-2-phenylindole (DAPI). All the antioxidant enzymes were expressed in cancer cell plasma.

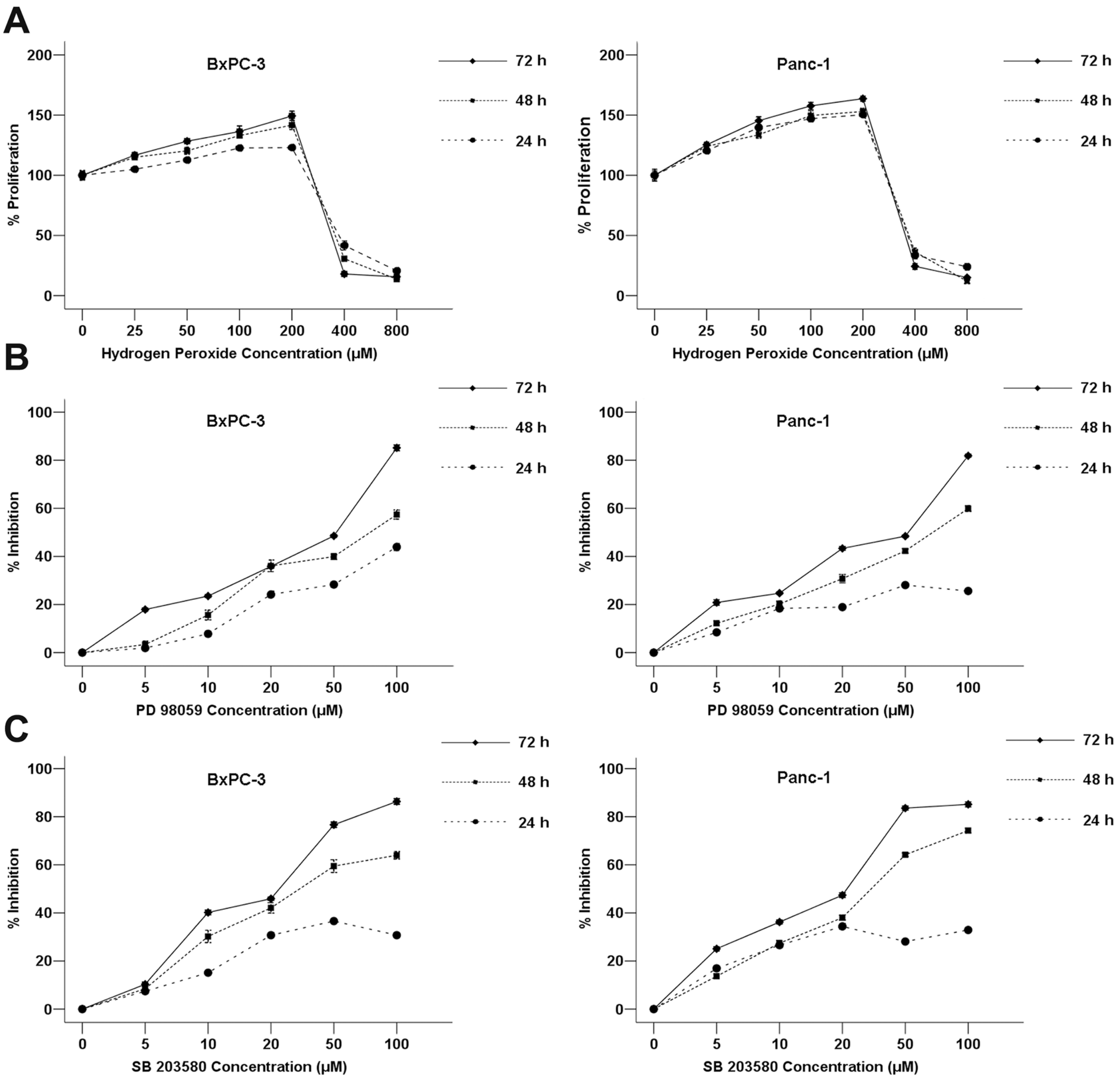

Supplementary Figure S2: The effect of H_2O_2 and/or MAPK inhibitors on cancer cell proliferation. A. Induction of cell proliferation of human pancreatic tumor cell lines BxPC-3 and Panc-1 by H_2O_2 at different concentrations (0, 25, 50, 100, 200, 400 and 800 μM). **B.** The inhibition ratio for BxPC-3 and Panc-1 PC cancer cell proliferation treated with increasing concentrations of ERK inhibitor, PD 98059 for 24 h, 48 h, or 72 h. **C.** The inhibition ratio for BxPC-3 and Panc-1 PC cancer cell proliferation treated with increasing concentrations of p38 MAPK inhibitor, SB 203580 for 24 h, 48 h, or 72 h.
